# Perceived delivery of essential yoga properties within in-person and remote weight loss maintenance interventions

**DOI:** 10.1371/journal.pone.0300105

**Published:** 2024-03-07

**Authors:** Sally A. Sherman, Tyler D. Quinn, Beth C. Bock, Tosca D. Braun, Jessica L. Unick

**Affiliations:** 1 Department of Health and Human Development, University of Pittsburgh, Pittsburgh, PA, United States of America; 2 Department of Epidemiology and Biostatistics, West Virginia University, Morgantown, WV, United States of America; 3 Department of Psychiatry and Human Behavior, Department of Behavioral and Social Sciences, Centers for Behavioral and Preventative Medicine, The Miriam Hospital, Brown University, Providence, RI, United States of America; 4 Department of Psychiatry and Human Behavior, Centers for Behavioral and Preventative Medicine, The Miriam Hospital, Brown University, Providence, RI, United States of America; 5 Department of Psychiatry and Human Behavior, Weight Control and Diabetes Research Center, The Miriam Hospital, Brown University, Providence, RI, United States of America; Utah State University, UNITED STATES

## Abstract

**Objectives:**

While previous research has utilized remote delivery of yoga interventions, no research has specifically interrogated the effectiveness of remote yoga intervention delivery. In this secondary analysis of weight-maintenance trial data, we examined participant perceptions of essential yoga properties across in-person and remote formats, hypothesizing that perceptions would not differ following remote delivery.

**Methods:**

24 women with overweight or obesity (34.6±4.1 kg/m^2^, 48.2±9.9 years) received a 12-week Iyengar yoga intervention (2x/week) following a 3-month behavioral weight loss program. Of 23 participants who completed follow-up questionnaires, 12 received the planned in-person intervention and 11 received a remote intervention (delivered live) due to the COVID-19 pandemic. The Essential Properties of Yoga Questionnaire (EPYQ) was completed online by participants and by the instructors to measure the perceptions of the relative emphasis placed on the essential components of the yoga intervention via 14 subscales. Linear regression models were used to compare perceptions of each EPYQ dimension across in-person and remote delivery methods, as well as between participants and instructors, independent of delivery method.

**Results:**

13 of the 14 subscales did not differ between delivery modalities (*p*>0.05). Participants perceived more individual attention within in-person yoga (*p* = 0.003). For both delivery methods, instructors perceived breathwork, restorative postures, and body locks to be incorporated to a lesser degree compared to participants (*β* = -1.28, *p* = 0.003; *β* = -1.57, p = 0.019; *β* = -1.39, *p* = 0.036; respectively). No other significant differences across the participant and instructor scores were observed.

**Conclusions:**

Findings provide preliminary support for the use of live remote delivery of yoga, effectively communicating most essential yoga properties when compared to in-person classes. However, participants perceived more individual attention with in-person versus remote delivery; thus, future remote-based yoga interventions may benefit from providing additional individualized feedback.

## Introduction

Hatha yoga is an ancient Indian practice comprising spiritual, ethical, and physical aspects [1, 2] Modern variants such as Iyengar yoga commonly involve physical postures, meditation, and breathing techniques [1, 2] A growing body of literature shows that yoga impacts many physical and psychological factors, [3–5] making it an effective strategy for improving well-being across a variety of health domains [6]. Research also indicates that yoga interventions can improve obesity-related parameters such as body mass index, fat mass, and weight circumference [7]. However, there is limited evidence as to whether these improvements result from favorable changes in eating behaviors, increased physical activity, or increased muscle mass [7]. Additional hypothesized pathways through which yoga may influence weight is via improved sleep, [8] decreased stress, [9] pain reduction, [10, 11] and improved mood [12] Preliminary data also suggest that yoga may be an effective strategy for improving weight loss maintenance among women with overweight or obesity, [13] a group understudied within yoga research. Considering these beneficial effects of yoga, it is important to consider how best to deliver yoga interventions to more individuals while ensuring the essential dimensions of the intervention (e.g., postures, breathwork, body awareness) are maintained. The Essential Properties of Yoga Questionnaire (EPYQ) quantifies and describes these yoga dimensions present within an intervention [14].

The COVID-19 pandemic resulted in many behavioral interventions experimenting with live remote formats to deliver intervention content; however, research examining how these formats (in-person versus remote) differ in feasibility, participant understanding of the intervention materials, and effectiveness of the instruction is limited. One previous study did examine differences in delivery of a 12-week strength and flexibility exercise intervention across in-person and remote formats [15]. No differences in intervention effectiveness in reducing low back pain were observed across the delivery methods [15]. Another study recently found that the exercise intensity of yoga was equivalent across both remote and in-person delivery formats regardless of practitioner proficiency [16]. While this research suggests that remote exercise intervention delivery may be effective in some cases, more research is necessary to fully understand the impact of delivery format in the context of yoga interventions. Specifically, it is unclear if participant or instructor perceptions of the yoga dimensions covered within the intervention differ across delivery methods. Importantly, the need for data examining the feasibility and efficacy of remote delivery of yoga interventions was highlighted in a recent review [17].

To fill this knowledge gap, secondary analyses from a weight loss maintenance trial were conducted in which electronic questionnaires were used to 1) compare participant perceived delivery of essential yoga dimensions across in-person and remote formats (Aim 1) and 2) compare instructor and participant perceptions of these yoga dimensions independent of delivery method (Aim 2). We hypothesized that the delivery method of the intervention would not significantly impact participant perceptions of the yoga dimensions and that there would be no significant differences in these perceptions across the participants and instructors.

## Methods

This study was a secondary analysis of a larger study. The larger trial compared a 3-month yoga intervention to a contact-matched control condition (cooking and nutrition classes) following three months of standard behavioral weight loss treatment. This standard treatment was designed to produce a 1–2 pound per week weight loss facilitated by weekly 60-minute group-based classes, prescribed caloric restriction, and moderate exercise prescription. Additional study details and primary findings related to weight, psychosocial factors, program satisfaction, and intervention adherence have been reported previously [13]. Within that study, 24 women with overweight or obesity were randomized to receive a 12-week Iyengar yoga intervention (2x/week) following the weight loss program. This secondary analysis includes yoga participants who completed study questionnaires following the yoga intervention (*n* = 23) (Table 1).

**Table 1:** Participant characteristics by intervention delivery method.

	Total	In-Person Delivery	Remote Delivery	*p*-value
	*n* = 23	*n* = 12	*n* = 11	
Age	48.0±10.1	48.2±9.8	47.6±10.9	0.890
Gender				
Female	23 (100.0%)	12 (100.0%)	11 (100.0%)	
Height (cm)	161.9±6.0	162.9±4.7	160.9±7.3	0.430
Weight (kg)	86.3±12.9	91.2±12.3	81.0±11.8	0.055
BMI	33.1±4.6	34.6±4.4	31.3±4.4	0.085
Race				0.480
White	20 (87.0%)	11 (91.7%)	9 (81.8%)	
Not White	3 (13.0%)	1 (8.3%)	2 (18.2%)	
Ethnicity				0.590
Not Hispanic or Latino	20 (87.0%)	10 (83.3%)	10 (90.9%)	
Hispanic or Latino	3 (13.0%)	2 (16.7%)	1 (9.1%)	
Marital Status				0.400
Married	15 (65.2%)	7 (58.3%)	8 (72.7%)	
Divorced	2 (8.7%)	2 (16.7%)	0 (0.0%)	
Never married	4 (17.4%)	2 (16.7%)	2 (18.2%)	
Not married living with significant other	1 (4.3%)	0 (0.0%)	1 (9.1%)	
Other	1 (4.3%)	1 (8.3%)	0 (0.0%)	
Mean EPYQ score	3.5±0.6	3.6±0.4	3.3±0.7	0.240

Footnotes:

a) Data are presented as mean±SD for continuous measures, and *n* (%) for categorical measures.

b) Weight and BMI are presented as values at the start of the yoga intervention, not the start of the weight loss program.

c) Continuous variables were tested for differences across delivery groups using *t*-tests and categorical variables were tested using *Chi-squared* tests.

Written informed consent was obtained prior to enrollment, all study procedures were approved to be ethical and in alignment with the Declaration of Helsinki by The Miriam Hospital’s Institutional Review Board (#1244203–5), and this study was registered on Clinical-Trials.gov (NCT03799289). Study data collection was conducted between January 2019 and June 2020 and the anonymity of all participants was maintained on all study records using participant identification numbers.

### Yoga intervention

Iyengar yoga is a form of hatha yoga that focuses on breathing, postures, and meditation with the use of props (e.g., chairs, straps, blocks) to allow for correct postural placement and to reduce risk of injury. Based on oxygen consumption and metabolic equivalent response, Iyengar yoga is shown to have a lower energy expenditure than other styles of yoga [18] requiring continuously flowing movements paired with breath, such as Vinyasa [19]. As such, Iyengar was used in this study to avoid any potential confounding with aerobic physical activity. 60-minute group classes were held twice weekly. Instructors were two certified Iyengar yoga instructors with 13–15 years of experience. Classes consisted of the following: a brief warm-up (~5–7 minutes), a period of more intense poses (~35 minutes), a cool-down consisting of more relaxing poses (~3–7 minutes), breathwork (~7–10 minutes), and Savasana (~2–10 minutes). Participants were encouraged to engage in self-initiated yoga practices at home and were provided with resources for practicing yoga on their own. Tools provided for home practice included audio recordings, handouts of suggested poses with instructions, and weekly notecards helping them apply the principles of yoga to behaviors outside of the yoga practice.

Of the 23 participants who completed the follow-up questionnaires, 12 received the planned in-person yoga intervention and 11 received a remote intervention due to the COVID-19 pandemic. Therefore, the participant enrollment timing was the primary determining factor in which participants were in each group. The yoga intervention remained the same between delivery formats and remote classes were delivered live via videoconferencing software with participant cameras turned on. Thus, instructors were able to provide corrections and students were able to ask questions using this remote format.

### Essential Properties of Yoga Questionnaire (EPYQ)

Following completion of the yoga intervention, the EPYQ was administered to both participants and yoga instructors to measure their perceptions of the emphasis placed on various components of the intervention via 14 subscales (Table 2). The EPYQ, developed and validated by Park et al. in 2018, Groessl et al. in 2015, and Elwy et al. in 2014, is a questionnaire collecting respondent perceptions of how much a yoga instructor mentioned or included various dimensions of yoga in a single yoga class [14, 20, 21]. Each question asked the respondent to answer the question “How much did the instructor mention or include…” certain aspects of the practice. Responses range from 0 (not at all) to 4 (a very large amount). The EPYQ contains 62 questions across 14 dimensions: acceptance/compassion, breathwork, physicality, active postures, restorative postures, body locks (bandhas), body awareness, mental and emotional awareness (release), health benefits, individual attention, social aspects, spirituality, meditation and mindfulness, and yoga philosophy. The items within the EPYQ questionnaire were finalized from 81 to 62 items in an international online survey of n = 1299 across adult yoga students, instructors, and researchers. The reliability and validity of the final questionnaire was assessed in a sample of n = 144 yoga instructors and students (79% female, 36% 26–35 years old, 81% white). In this sample, all 14 dimensions had Cronbach’s alphas between 0.70 and 0.90, with an average across all dimensions of 0.93. An average interclass correlation coefficient (ICC) of 0.943 was observed across all dimensions with a range from 0.712 (yoga philosophy) to 0.982 (individual attention) [14].

**Table 2:** Comparison of EPYQ dimensions between delivery method and participants/instructors.

	Effect of Delivery Method^b^		Effect of Participant/Instructor^c^		Interaction^d^
	*β*±*SE*	*p*-value	*β*±*SE*	*p*-value	*p*-value
Acceptance / Compassion	-0.47±0.38	0.233	-0.17±0.70	0.813	0.816
Breathwork	-0.16±0.21	0.459	**-1.28±0.38**	**0.003**	0.918
Physicality	-0.22±0.30	0.476	0.25±0.55	0.651	0.965
Postures (Asanas)–Active	-0.22±0.31	0.490	0.17±0.57	0.774	0.790
Postures (Asanas)–Restorative	-0.12±0.34	0.724	**-1.57±0.62**	**0.019**	0.841
Body Locks (Bandhas)	-0.53±0.34	0.137	**-1.39±0.62**	**0.036**	0.977
Body Awareness	-0.00±0.27	0.993	0.53±0.49	0.290	0.815
Mental & Emotional Awareness	0.11±0.30	0.725	-0.57±0.54	0.309	0.792
Health Benefits	-0.62±0.37	0.109	-0.54±0.68	0.435	0.705
Individual Attention	**-1.03±0.29**	**0.002**	-1.00±0.53	0.074	0.968
Social Aspects	0.20±0.28	0.473	-0.08±0.51	0.872	0.343
Spirituality	-0.25±0.29	0.403	0.35±0.53	0.513	0.998
Meditation & Mindfulness	-0.14±0.37	0.719	0.17±0.69	0.810	0.650
Yoga Philosophy	-0.04±0.47	0.941	0.72±0.86	0.410	0.707

Footnotes:

a) Boldface text indicates significant results (*p*<0.05).

b) The reference category is the in-person delivery score where the Beta coefficient (*β*) indicates a difference between in-person and remote (remote score–in-person score).

c) The reference category is the participant scores where the Beta coefficient (*β*) indicates a difference between the participant and instructor scores (instructor score–participant score).

d) Interaction between the delivery method and participant/instructor.

e) All scores are on a scale from 1–5 where a higher score indicates that the respondent perceived the class to have more of that dimension.

This questionnaire was included as part of this trial to enhance scientific rigor so that the delivered yoga intervention could be more quantitively described. However, the necessary pivot from in-person to remote delivery of the yoga intervention also provided the unique opportunity for these secondary data analyses comparing delivery formats on these essential yoga properties; something that has not been previously examined. The EPYQ was designed for trained raters to measure, describe, and quantify the important dimensions in yoga interventions so that yoga is more adequately described within scientific manuscripts [14]. For this analysis, we examined whether participant and/or instructor perceptions of the EPYQ yoga dimensions differed when yoga was delivered in-person versus remotely. This also allowed for a novel comparison of instructor versus participant perceptions.

### Statistical analysis

**Fig 1:**
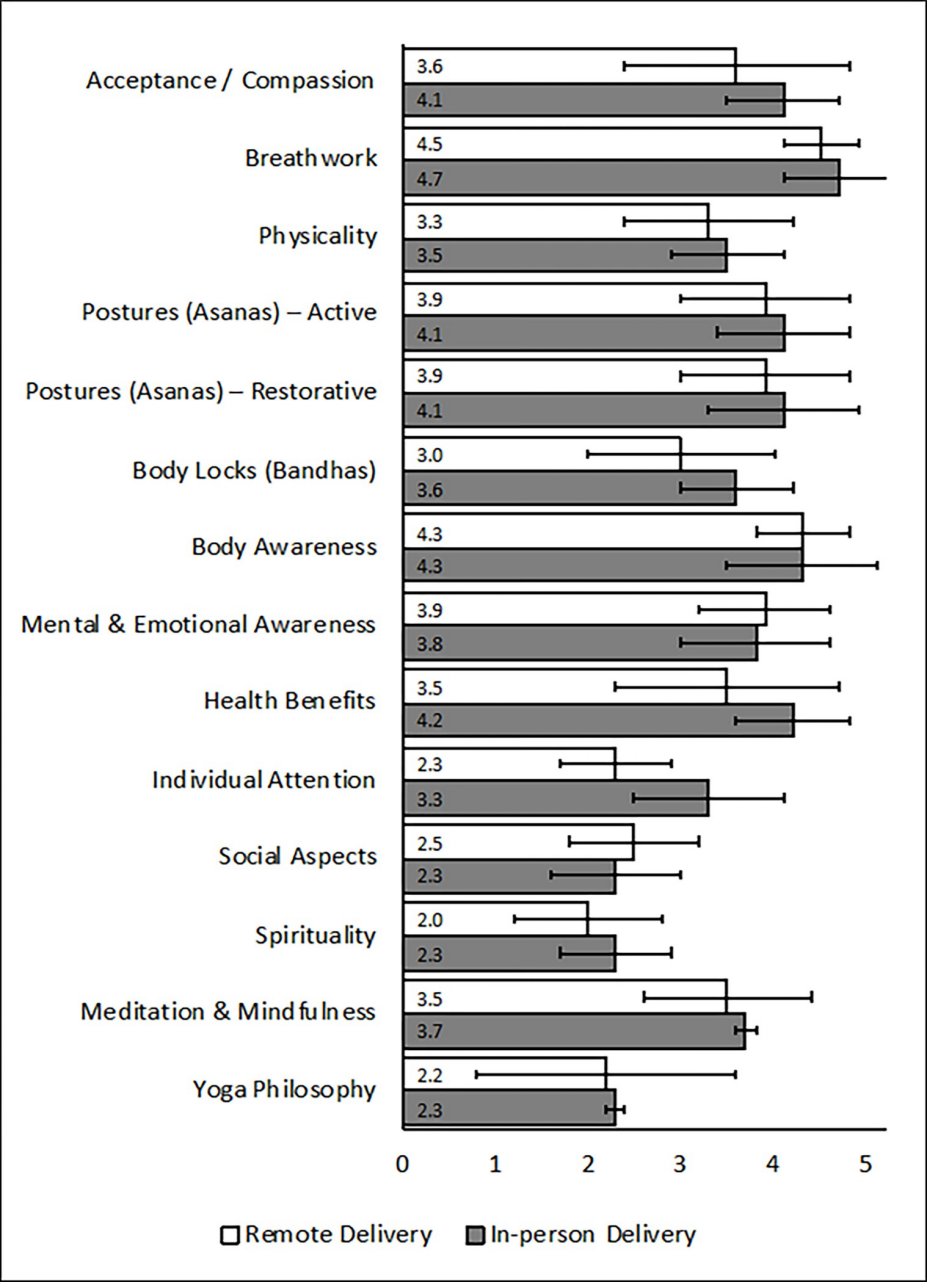
Comparison of EPYQ dimensions between participants and instructors. Footnotes: a) Values presented are the mean scores for the corresponding scale and delivery method. b) The error bars represent plus or minus one standard deviation from the mean score. c) * indicates a statistically significant difference between in-person and remote (*p*<0.05).

Participant demographics were summarized using descriptive statistics. All EPYQ sub-scales were approximately normally distributed and contained no apparent outliers. Next, a single linear regression model for each EPYQ subscale compared the scores across delivery methods (Aim 1) as well as between the participants and instructors and the interaction between these factors (delivery method x participant/instructor) (Aim 2). In-person was used as the reference category for the delivery method comparison where the Beta coefficient indicates a difference between in-person and remote (remote score–in-person score). Similarly, the participant score was used as the reference category to the participant/instructor comparison where the Beta coefficient indicates a difference between the participant and instructor scores (instructor score–participant score). A significant interaction should be interpreted to mean that the difference between the instructor and participant scores are different between delivery methods. Finally, comparisons were made using independent sample t-tests for each EPYQ subscale score between in-person and remote delivery groups for participants to examine the impact of delivery method on participant perceptions of EPYQ dimensions (Fig 1). All analysis were completed using STATA v.17 and the alpha level was set to 0.05.

## Results

Table 1 presents the participant demographics. Overall, the intervention participants were 48.2±9.9 years old, were all female, had a BMI = 33.1±4.6 kg/m^2^, and were 87% White. No significant differences between the in-person and remote delivery groups were observed.

Fig 1 presents the EPYQ subscale scores for participants by delivery method (Aim 1). Individual attention was significantly lower in remote (2.3±0.6) versus in-person delivery (3.3±0.8, *β* = -1.03, *p* = 0.002). However, no other significant differences between delivery methods were observed (*p*>0.05).

Table 2 presents comparisons of EPYQ scores by delivery method, participants versus instructors, and the interaction (Aim 2). For both delivery methods, instructors perceived breathwork, restorative postures, and body locks to be incorporated to a lesser degree compared to participants (*β* = -1.28, *p* = 0.003; *β* = -1.57, *p* = 0.019; *β* = -1.39, *p* = 0.036; respectively). No other significant differences across the participant and instructor scores were observed. The differences in the EPYQ dimensions perceived by participants versus instructors were not significantly modified by the delivery method. This can be observed by the fact that no significant delivery method x participant/instructor interaction effects were found for any EPYQ subscale (*p*>0.05 for all).

## Discussion

Participant perceptions of the yoga dimensions delivered across in-person and live remote delivery methods were not significantly different. However, participants did perceive greater individual attention with in-person delivery. Additionally, instructor perceptions of the various yoga dimensions delivered were largely similar to the perceptions of the participants, independent of the delivery method (in-person versus remote). Taken together, the current study findings were supportive of our hypotheses and provide preliminary support for the use of remote delivery of yoga interventions among women with overweight or obesity.

As assessed via Aim 1, study findings indicate that most essential yoga dimensions can be similarly communicated during live yoga classes delivered remotely and in-person among women with overweight/obesity. This finding is in agreement with the limited previous research comparing in-person versus remote delivery of exercise or yoga interventions. For example, one comparable study found no difference in exercise intervention effectiveness across in-person or remote delivery formats on changes in low back pain [15] Another study recently observed that in-person versus remote delivery of a yoga intervention did not significantly impact the exercise intensity experienced by the participants regardless of practitioner proficiency [16]. While these studies did not specifically measure the essential yoga dimensions as in the current study, they do provide additional preliminary data to support the remote delivery of exercise interventions.

While most yoga dimensions were not perceived differently across delivery methods, our results suggest that participants may perceive more individual attention with in-person versus remote delivery. While not tested directly here, this differential perception in individual attention may be due to a lack of perceived instructor eye contact, physical presence, or opportunity for postural adaptations using physical contact during the remote instruction. Additionally, interpersonal interaction between the instructor and participants before and after the class may be limited during the remote delivery of the class. Considering this, when teaching live, remote-based yoga classes, instructors may want to consider providing more individualized feedback to participants, individualized verbal cues/corrections, and provide opportunities to engage participants both before and after class within the virtual environment. As previously reported in the primary outcome paper, in-person and remote cohorts had similar program satisfaction ratings and high rates of attendance (82.2±26.8% versus 69.6±21.9% respectively, *p* = 0.22) [13]. This, coupled with the current findings, suggest that remote delivery of yoga may be a potential strategy for effectively delivering interventions while also maintaining treatment fidelity. While these initial findings support the use of remote interventions, future effectiveness studies are certainly warranted given the lack of robust data.

To our knowledge, this was the first study to compare instructor versus participant perceptions of yoga classes. In support of our Aim 2 hypothesis, instructors and participants largely perceived the delivery of the EPYQ dimensions in this Iyengar yoga intervention to be similar. However, when differences did occur (breathwork, restorative postures, and body locks), the instructors perceived the level of dimension delivery as lower than the participants. This may relate in part to the differential knowledge of yoga among participants versus instructors. For instance, one possible reason for increased perception of breathwork among participants versus instructors is that the former may interpret simple attention to or cueing of the breath throughout class as “breathwork,” whereas instructors may more formally define breathwork as standalone pranayama exercises. Similarly, restorative postures and body locks entail very specific definitions and techniques. Given their expert knowledge of yoga, instructor perceptions of such practices are likely to be more accurate than participant appraisals. However, the difference in the perceptions between instructors and participants did not depend on the delivery method (i.e., no significant interactions were observed). Considering this, the results largely indicate that the perceived delivery of yoga dimensions by instructors are effectively being experienced by the participants in the class regardless of the delivery method (in-person or remote).

### Strengths and limitations

This study was strengthened most notably by its novel measurement of EPYQ yoga dimensions across remote and in-person delivery as well as across intervention participants and instructors which supported unique and informative comparisons. This study was however limited in its sample size and statistical power as it was a secondary analysis of only one arm of a larger study. In addition, the lack of diversity in participant demographics limits generalizability to other populations. Lastly, this study measured the EPYQ in a questionnaire format among yoga instructors and intervention participants. The measure was designed for use by raters specifically trained in understanding each essential property of yoga. It is not validated for less trained populations (e.g., novice yoga students) which may limit the internal validity of the findings. However, this study was interested in the participant’s perceptions of the presence of each property and not necessarily the actual presence of said property, or other associated factors, such as their deeper comprehension thereof or proficiency in performing related techniques.

## Conclusions

While future, more rigorous examination is needed, these preliminary findings suggest live remote-based yoga interventions may be a viable delivery method that effectively communicates the most essential yoga dimensions compared to in-person classes. However, future remote-based yoga interventions may benefit from more individualized feedback to participants, individualized verbal cues/corrections, and by providing opportunities to engage participants both before and after class within the virtual environment.
